# A decade to wait: Update on the average delay to diagnosis for endometriosis in Aotearoa New Zealand

**DOI:** 10.1111/ajo.13836

**Published:** 2024-05-22

**Authors:** Katherine Ellis, Rachael Wood

**Affiliations:** ^1^ Department of Chemical and Process Engineering University of Canterbury Christchurch New Zealand; ^2^ Endometriosis New Zealand Christchurch New Zealand; ^3^ Biomolecular Interaction Centre University of Canterbury Christchurch New Zealand

**Keywords:** endometriosis, New Zealand, delay to diagnosis, diagnostic delay

## Abstract

Endometriosis is a common condition with varying delays from symptom onset to diagnosis reported internationally. In New Zealand, the previously accepted average delay to diagnosis was 8.6–8.7 years. An online survey completed by the largest cohort of self‐reported New Zealand‐confirmed endometriosis patients (*n* = 1024) for the collection of delay to diagnosis was conducted in September and October of 2023. The results revealed an average delay of 9.7 ± 7.1 years overall, with a significantly longer delay in the North Island than in the South. This study identifies potential factors for future research that may influence diagnostic delays in New Zealand.

## INTRODUCTION

Delays from symptom onset to diagnosis for endometriosis, a condition where extrauterine endometrial‐like implants are found,^1^ have been identified as a potentially significant factor in higher financial costs, number of symptoms, and healthcare utilisation requirements,^2^ although the extent to which this is a factor in New Zealand (NZ) is unknown due to a lack of data. Reports of the delay to diagnosis internationally vary, with a report of average delays of 6.6 years for Ibero‐America, 10.4 years for Austria‐Germany,^3^ 5.4 years in Canada,^4^ and 8.6 and 8.7 years in prior NZ studies.^5^, ^6^ In NZ, small sample studies also indicate that Māori and Pasifika patients may face longer delays of 11.6 ± 7.8 and 12.4 ± 6.2 years respectively.^7^ Key features that feed into these delays are low knowledge of endometriosis existence at symptom onset, normalisation of symptoms, dismissal of suffering,^5^, ^8^, ^9^ the need for further primary practitioner education,^9^, ^10^, ^11^ and healthcare system type with longer delays identified in state‐funded healthcare systems.^12^ The risk of endometriosis is consistently associated with lower birth weight, earlier age at the onset of menstruation, lower body mass index, shorter menstrual cycles and lower parity^13^ and an estimated 10% of people presumed female at birth are thought to be affected by the condition.^14^

## MATERIALS AND METHODS

### Survey design and approval

The delay to diagnosis from symptom onset data presented is part of a larger survey distributed in September and October of 2023. There were 1262 responses to the survey, which focused on the research priorities of endometriosis patients and their support networks (completion rate 90.1%). Among the 1262 responses, 1024 individuals had self‐reported surgically or radiologically confirmed endometriosis (completion rate 92.1%), and they self‐reported their delay to diagnosis in years from symptom onset to confirmation of the condition in response to the question: ‘How long was it from when you started to have symptoms of endometriosis to when you received your confirmed diagnosis? eg X years’. When a range of time was given in response, the mid‐point of the range was recorded, eg 5.5 years for a report of ‘5–6 years’. The design and distribution of the survey was approved by the Board of Governance of Endometriosis New Zealand, and ethics approval was obtained from the University of Canterbury Human Research Ethics Committee (Ref: HREC 2023/92‐LR/PS) following consultation with the Ngāi Tahu Consultation and Engagement Group.

### Recruitment

Recruitment for this survey was predominantly through social media with snowball recruitment by the authors and Endometriosis New Zealand who shared the study information and anonymous survey link through their networks, which could then be further distributed by interested members of the public. Endometriosis New Zealand also distributed the invitation to participate through their opt‐in mailing list composed of endometriosis patients and their support networks. All participant information was self‐reported and not validated using any other sources to maintain participant anonymity.

Participants were informed at the information screen that they had to be over the age of 18, and reside in NZ. While participants cannot be confirmed to be NZ residents, only 0.5% of participants did not indicate the region of NZ they lived in. It was therefore assumed that participants acting in good faith would be unlikely to complete the survey while residing in a different country. To try and ensure the representation of hard‐to‐reach groups, including individuals who identify as Māori, Pasifika, and part of the Rainbow community, organisations associated with these groups were contacted with the survey information with the request to share the study invitation.

### Statistical analysis

Statistical significance was set at *α* < 0.05. Data were analysed with GraphPad (Version 9). The null hypothesis in all cases was that means were equal. Shapiro–Wilk tests were used to determine the normality of the data. Since data were non‐normally distributed, Kruskal–Wallis tests with Dunn's multiple comparison post‐hoc, or Mann–Whitney tests were used for statistical reporting. Differences in features of the groups reporting radiologically and surgically confirmed diagnoses were conducted using Pearson's χ^2^ tests. All reported values are the mean ± the standard deviation unless otherwise stated.

## RESULTS

### Participant cohort

Of the 1024 survey participants who reported confirmed endometriosis (Table 1), 955 (93.3%) reported being surgically diagnosed, while 69 (6.7%) reported being radiologically diagnosed, through imaging modalities such as magnetic resonance imaging and transvaginal ultrasound.^15^ Between these two groups, there were no significant differences in age or gender identity. There were significantly more Asian participants (*P* < 0.01), as well as more people from Auckland (*P* < 0.01), and Bay of Plenty (*P* < 0.05) regions, and semi‐rural areas (*P* < 0.05) among the group reporting radiologically confirmed endometriosis. More NZ European participants (*P* < 0.01), as well as people from the Wellington region (*P* < 0.05) and urban areas (*P* < 0.05) were among the group reporting surgically confirmed diagnoses (*P* < 0.01).

**Table 1:** Demographics of endometriosis patients reporting confirmed diagnoses *N* = 1024

	Surgically confirmed *n* = 955	Radiologically confirmed *n* = 69
Age		
18–24	128 (13.4%)	8 (11.6%)
25–34	402 (42.1%)	23 (33.3%)
35–44	265 (27.7%)	24 (34.8%)
45–54	131 (13.7%)	13 (18.8%)
55+	28 (2.9%)	1 (1.4%)
Gender identity		
Female	937 (98.1%)	67 (97.1%)
Male	1 (0.1%)	
Trans male	1 (0.1%)	
Gender diverse		1 (1.4%)
Non‐binary	14 (1.5%)	
Takatāpui	1 (0.1%)	
Agender		1 (1.4%)
Ethnicity (multiple selections possible)		
NZ European	828 (86.7%)	51 (73.9%)
Māori	140 (14.7%)	7 (10.1%)
Asian	18 (1.9%)	5 (7.2%)
Pacific Peoples	25 (2.6%)	4 (5.8%)
Latin American	8 (0.8%)	2 (2.9%)
African	5 (0.5%)	
Middle Eastern	5 (0.5%)	
Residence		
Rural	81 (8.5%)	7 (10.1%)
Semi‐rural	112 (11.7%)	14 (20.3%)
Urban	755 (79.1%)	47 (68.1%)
Region of residence		
Northland/Te Tai Tokerau	19 (2.0%)	1 (1.4%)
Auckland/Tāmaki‐Makau‐Rau	229 (24.0%)	27 (39.1%)
Bay of Plenty/Te Moana‐a‐Toi	45 (4.7%)	7 (10.1%)
Waikato	96 (10.1%)	6 (8.7%)
Taranaki	16 (1.7%)	
Gisborne/Te Tairāwhiti	4 (0.4%)	1 (1.4%)
Hawke's Bay/Te Matau‐a‐Māui	39 (4.1%)	2 (2.9%)
Manawatū‐Whanganui	43 (4.5%)	1 (1.4%)
Wellington/Te Whanga‐nui‐a‐Tara	139 (14.6%)	4 (5.8%)
Tasman/Te Tai‐o‐Aorere	14 (1.5%)	
Nelson/Whakatū	17 (1.8%)	3 (4.3%)
Marlborough/Te Tau Ihu‐o‐te‐Waka	5 (0.5%)	
West Coast/Te Tai Poutini	7 (0.7%)	
Canterbury/Waitaha	200 (20.9%)	11 (15.9%)
Otago/Ōtākou	51 (5.3%)	5 (7.2%)
Southland/Murihiku	26 (2.7%)	1 (1.4%)

There were participants from all regions of NZ. Only five of the 16 regions had participant proportions more than 1% different from the population proportion. The under‐represented regions were Northland (2.0% in the sample, 3.9% of the population), Auckland (25.1%, 33.3%), and the Bay of Plenty (5.1%, 6.8%), while the over‐represented regions were Wellington (14.0%, 10.5%), and Canterbury (20.7%, 12.8%).^16^

### Delay to diagnosis

**Figure 1:**
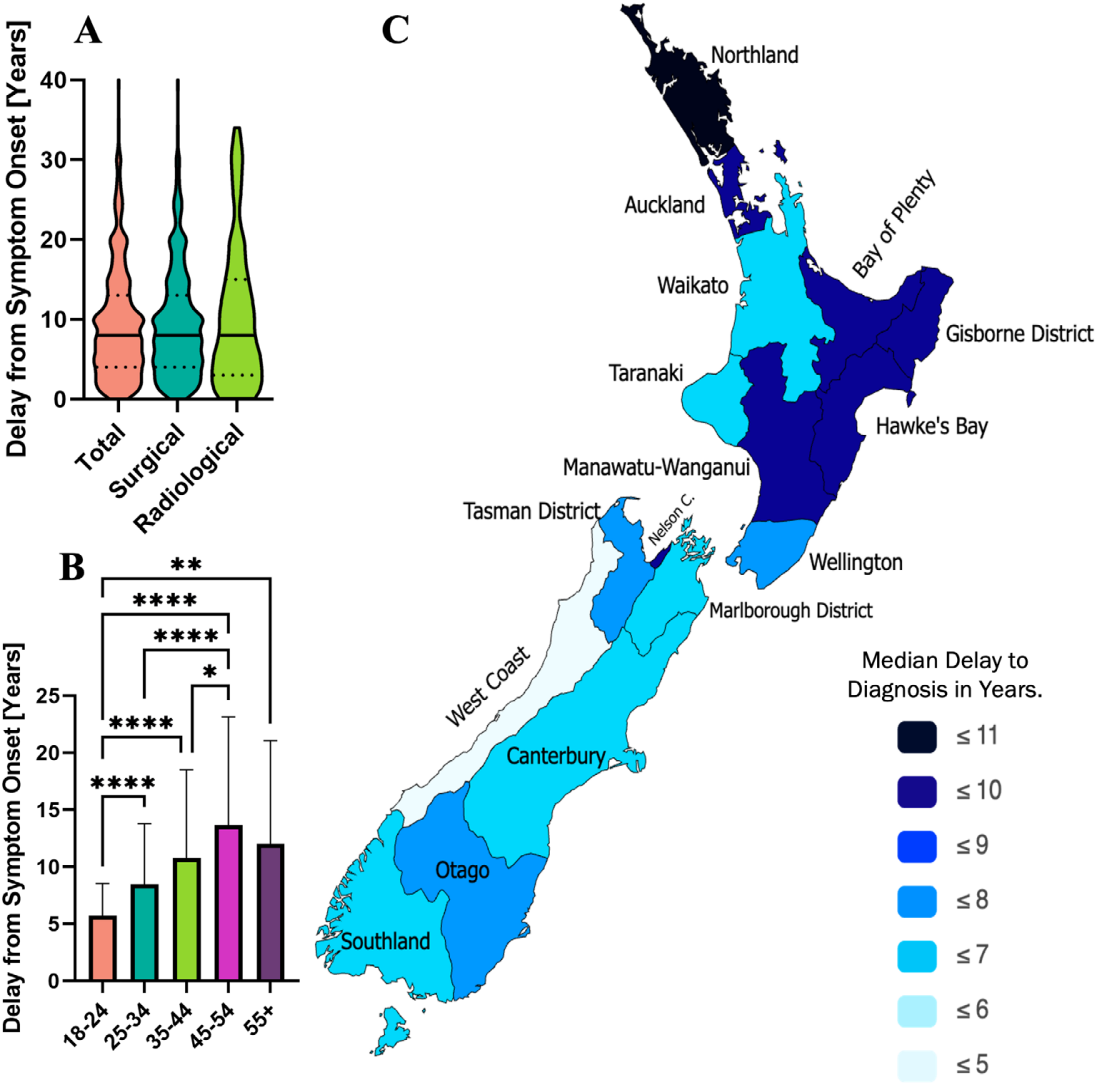
Delay to diagnosis data with average ± standard deviation unless stated otherwise for (A) violin plots for the overall cohort (*n* = 1013), for those reporting surgically confirmed endometriosis (*n* = 938) and those reporting radiologically confirmed endometriosis (*n* = 69), solid black lines represent median, dotted black lines indicate the interquartile range, (B) aged 18–24 (*n* = 135), 25–34 (*n* = 421), 35–44 (*n* = 282), 45–54 (*n* = 141), and 55+ (*n* = 28). (C) Map of New Zealand regions displaying spatial distributions of median delay to diagnosis made in paintmaps.com for patients residing in Northland (*n* = 20), Auckland (*n* = 253), Bay of Plenty (*n* = 51), Waikato (*n* = 101), Taranaki (*n* = 16), Gisborne (*n* = 5), Hawke's Bay (*n* = 38), Manawatū‐Whanganui (*n* = 44), Wellington (*n* = 140), Tasman (*n* = 14), Nelson (*n* = 20), Marlborough (*n* = 5), West Coast (*n* = 7), Canterbury (*n* = 206), Otago (*n* = 56), and Southland (*n* = 26) regions. (F) Statistical significance is signified by *P < 0.05, **P < 0.01, ***P < 0.001, ****P < 0.0001 derived from the Kruskal–Wallis test with Dunn's multiple comparison post‐hoc test.

Previously, the largest NZ cohort (*n* = 615) estimation of the average delay from symptom onset to diagnosis was 8.7 years;^6^ however, the current survey indicates that the average may be higher, with an average delay of 9.6 ± 7.1 years (*n* = 1024, Fig. 1A). The delay to the average diagnoses for those reporting radiological diagnosis was not significantly longer than for those self‐reporting surgically confirmed endometriosis, at 10.4 ± 8.9 years and 9.5 ± 6.9 years respectively.

#### Variation with age

When this delay to diagnosis is examined by age (Fig. 1B), there is a general trend of increasing delay to diagnosis in older age groups, with an average delay of 5.7 ± 2.8 years for the 18‐24‐year‐olds, 8.5 ± 5.3 for 25–34s, 10.8 ± 7.8 for 35–44s, 13.6 ± 9.5 for 45–54s, and 12.0 ± 9.1 for the 55+ cohort. The delay for 18–24s was significantly lower than every other age group (*P* < 0.0001 for 25–34, 35–44, and 45–54 age groups, and *P* = 0.0043 for 55+), the 25–34s delay to diagnosis was significantly lower than the 45–54s (*P* < 0.0001), and the 35–44s delay was significantly lower than the 45–54s (*P* = 0.0479).

The participants in the 18–24 age group had a significantly lower proportion of reporting surgically confirmed diagnoses of endometriosis than the 25–34, 35–44 (*P* < 0.001), 45–54 and 55+ age groups (*P* < 0.01). Similarly, the 18–24 age group were significantly more likely to report having a clinically suspected diagnosis of endometriosis than the 25–34, 35–44, 45–54 (*P* < 0.001) and the 55+ age groups (*P* < 0.01). The 25–34 age group had a significantly higher proportion of clinically suspected endometriosis than the 45–54 age group (*P* < 0.05). There were no differences in the proportions of age groups reporting surgically confirmed or radiologically confirmed endometriosis diagnoses.

#### Variation with ethnicity and gender identity

There were no significant differences in delay from symptom onset to diagnosis among ethnic groups with an average delay of 9.4 ± 6.8 years for participants who identified as only NZ European, an average of 9.7 ± 7.2 for those who identified as Māori, an average of 10.1 ± 6.6 for Pasifika, and an average of 8.1 ± 5.4 for Asian endometriosis patients. For participants who identified as non‐cis‐gender and reported confirmed endometriosis diagnoses, the average delay to diagnosis was 9.1 ± 5.7 years.

#### Variation by region

There was an increased diagnostic delay among participants presently residing in semi‐rural compared to rural areas (10.8 ± 7.5 and 8.3 ± 6.2 years respectively, *P* = 0.0402), but no significant differences between the rural and semi‐rural participants and urban participants, with an average delay of 9.6 ± 7.1 years. There were no significant differences in the delays of participants residing in the 16 different regions of NZ.

The median delays to diagnosis were plotted on a map of NZ (Fig. 1C). When assessed with a Mann–Whitney test, it was found that the average delay to diagnosis was 10.0 ± 7.1 years in the North Island (*n* = 668), and 8.8 ± 6.9 in the South Island (*n* = 334) which is significantly shorter (*P* = 0.0051). When comparing the North and South Island groups, there were no differences in gender identity or diagnosis type. The North Island participants included a significantly higher proportion of 35–44‐year‐olds (*P* < 0.05), Pasifika (*P* < 0.001), urban (*P* < 0.05), and a lower proportion of NZ European (*P* < 0.01), and semi‐rural participants (*P* < 0.01).

## DISCUSSION

This study identified a longer than previously reported average delay from symptom onset to diagnosis in NZ, with an increasing delay with age, which may relate to the lower proportion of people reporting confirmed diagnoses in younger cohorts. This study also identified longer average and median delays in the North Island compared to the South Island. In NZ, 76.5% of the population is in the North Island,^16^ and of the 74 mapped specialists or clinics with the ability to provide endometriosis care in the publicly available database HealthPages, 91.9% are in the North Island.^17^ Therefore, the availability of individuals and clinics alone does not explain the difference in delays between the two islands. While not all specialists are necessarily present in this database, it does represent the presence of specialist care in a publicly available resource that is readily available for patients to locate care.

To assess the relative presence of specialists in each region, the proportion of specialists and clinics in each region was divided by the proportion of the population living in that region.^16^, ^17^ When the presence of specialists and clinics is broken down by region, Auckland (1.87), Taranaki (1.10), Wellington (1.41), Marlborough (1.35), Nelson (1.27), and Tasman (2.38) regions have a proportion of specialists (or clinics) larger than their proportion of the nation's population, while the Waikato (0.81), Bay of Plenty (0.20), Manawatū‐Whanganui (0.54), and Canterbury (0.21) regions had a lower proportion of specialists for the population proportion.^16^, ^17^ The remaining six regions had no specialists or clinics registered on HealthPages.^17^ From the present data set, there is no clear pattern between the distribution of the specialist services registered on HealthPages, and the delay to diagnosis. For example, Auckland has one of the highest specialist‐to‐population proportions with 62.2% of the registered specialists and clinics for 33.3% of the population, but also a median delay of a decade, while Canterbury has one of the poorest ratios, but a median delay of seven years. Canterbury is over‐represented in this study's sample relative to Auckland, which is likely due to the presence of both the research team and Endometriosis New Zealand in Canterbury. Therefore, you would expect the over‐representation of Canterbury, and the under‐representation of Auckland, to skew the diagnostic delay data toward the generally more specialist‐scarce, but also less delayed experiences of Canterbury. The mismatch between the availability of specialists and diagnostic delay is an area that requires further research to identify the reasons why greater specialist care availability does not necessarily reduce diagnostic delay.

### Limitations

#### Current geographical location

One limitation with the finding of a longer delay to diagnosis in current residents of the North Island compared to the South Island is that this dataset does not capture complexities such as moving between regions during or after the period of delay to diagnosis, and how that influences the overall regional delays. These complexities could be an area for future research, along with an investigation of how access to, and availability of, local public and private specialist care, may influence the overall patterns of diagnostic delay in NZ. This may allow for the identification of factors within the health system which will facilitate faster diagnosis of endometriosis. Identifying these key features is increasingly important as in 2022 the NZ health system shifted from a regional to centralised model,^18^ creating a potential window of opportunity for the institution of systematic changes that emphasise factors that may already be allowing for shorter delays in some areas of NZ, a desire already highlighted by a cohort of 50 NZ endometriosis patients.^8^ Patient‐centred research should be continually conducted to ensure future solutions and plans are co‐designed with patients of all backgrounds to work to ensure these changes do not replicate or worsen existing healthcare equity issues in NZ.^19^, ^20^, ^21^

#### Recall bias

Another limitation in this study includes that the collection of diagnostic delay in this study is subject to recall bias, as individuals had to remember the time in their life when symptom onset occurred, and how long from that time it was until they received their confirmed diagnosis. This bias is particularly likely to be present as the delays reported are substantial, requiring the recall of a period of a decade on average in response to the question. This study was anonymous, therefore these reported delays cannot be validated by the use of clinical records, and so are subject to the individual participants accurately recalling when their endometriosis symptoms began. The question also refers to ‘symptoms of endometriosis’ and not a specific symptom, such as pelvic pain, which means participants need to have identified the endometriosis symptom they first experienced and identify that symptom as endometriosis‐related. This can be particularly complex as prior studies in NZ have highlighted a wide range of symptoms patients have associated with endometriosis.^5^, ^6^

#### Recruitment method

This study was predominantly recruited for through a patient organisation, and there is research to indicate this method of recruitment (*n* = 291) can produce cohorts with significantly longer delays to diagnosis, and younger age at symptom onset than recruitment through secondary (*n* = 63) and tertiary care (*n* = 135).^22^ Therefore, the current study may be skewed toward longer delays; however, this recruitment methodology is consistent with the other large cohort study in NZ.^6^
